# Working Memory Maintenance Modulates Serial Dependence Effects of Perceived Emotional Expression

**DOI:** 10.3389/fpsyg.2019.01610

**Published:** 2019-07-11

**Authors:** Gaoxing Mei, Shiyu Chen, Bo Dong

**Affiliations:** ^1^School of Psychology, Guizhou Normal University, Guiyang, China; ^2^Department of Psychology, School of Education and Public Administration, Suzhou University of Science and Technology, Suzhou, China

**Keywords:** serial dependence, emotional expression, face perception, working memory, positive aftereffect

## Abstract

The stability of face perception is vital in interpersonal interactions. Recent studies have revealed the mechanism of the stability in the perception of stable attributes of faces (such as facial identity) by serial dependence, a phenomenon in which perception of current stimuli is pulled toward recently viewed stimuli. However, whether serial dependence of perceived emotional expression (a changeable attribute of faces) exists remains controversial, and its exact nature has not been examined yet. To address these issues, we used the methods of constant stimuli and two-interval forced choice tasks in three psychophysical experiments. Participants compared two successive facial expressions selected from a continuum with 50 morphed faces ranging from sad to happy. Experiment 1a and 1b showed that a perceived facial expression pulled toward previously seen facial expressions (i.e., a significant serial dependence effect), independent of response instructions. Furthermore, a stronger serial dependence effect was found when the first facial expression was retained in working memory for a longer delay duration (Experiment 2), and yet a weaker serial dependence effect was observed when a longer delay between decision and response was performed (Experiment 3). These findings indicate that serial dependence facilitates the stability of facial expression perception and is modulated by working memory representations.

## Introduction

Past visual experiences shape our current perception toward the visual world. On the one hand, it is well established that the visual system maintains high sensitivity to changes in the environment through visual adaptation, a phenomenon in which prolonged exposure to visual stimuli leads to negative aftereffects (for reviews, see Kohn, 2007; Webster, 2011, 2015). For example, when exposed to a sad facial expression for several seconds, observers perceived current faces as less sad (Hsu and Young, 2004; Ying and Xu, 2017; Palermo et al., 2018). On the other hand, recent studies have demonstrated that the visual system also maintains a stable and continuous perception in the noisy visual environment over time through serial dependence, a phenomenon in which the perception of current stimuli is pulled toward recently viewed stimuli (i.e., positive aftereffects) (Fischer and Whitney, 2014; Liberman et al., 2014; Kiyonaga et al., 2017). For example, perceived orientations of gratings were systematically biased toward recently viewed orientations in an orientation judgment task (Fischer and Whitney, 2014).

Serial dependence effects have frequently been reported for basic visual attributes, such as orientation (Fischer and Whitney, 2014; Liberman et al., 2016; Manassi et al., 2017), numerosity (Corbett et al., 2011; Cicchini et al., 2014; Fornaciai and Park, 2018), motion (Alais et al., 2017), size (Alexi et al., 2018), and position (Papadimitriou et al., 2015; Papadimitriou and Snyder, 2017; Manassi et al., 2018). Serial dependence has been considered a reflection of the Continuity Field: a spatiotemporal region where similar visual stimuli are integrated (Fischer and Whitney, 2014; Liberman et al., 2018).

Human faces, as naturally complex stimuli, have both stable attributes (e.g., gender of faces) and changeable attributes (e.g., emotional expression of faces). Serial dependence effects have recently been observed in various aspects of stable attributes of faces, such as facial identity (Liberman et al., 2014), facial gender (Taubert et al., 2016a), and facial attractiveness (Taubert et al., 2016b; Xia et al., 2016; Kok et al., 2017). However, inconsistent results have been found for a changeable attribute of faces – facial emotional expression. Using a sequence of morphed facial stimuli created in the two dimensions of gender (a stable attribute) and expression (a changeable attribute), Taubert et al. (2016a) presented a string of images to participants sequentially (the method of constant stimulus), and instructed them to perform two responses: the face is female or male and happy or sad. The results showed the opposite effects for the perception of these two attributes: clear positive serial dependencies for gender and well-known negative aftereffects for expression. They reasoned that there was no positive serial dependence of changeable attributes like facial expressions. However, using the method of adjustment (MOA) task where participants were instructed to adjust a variable stimulus to match a target stimulus, Liberman et al. (2018) most recently found that perception of a facial expression was biased toward recently viewed facial expressions (i.e., a positive serial dependence effect). Given these inconsistent findings, our first aim (Experiment 1) was to replicate Liberman et al. (2018)’s experiments using a two-interval forced choice (2IFC) task rather than their original MOA task, in order to investigate whether serial dependence of perceived facial expression could occur. In a 2IFC task, participants are sequentially presented with two alternative stimuli (e.g., faces) in two intervals, and are instructed to indicate which of the two stimuli is perceived to be the correct option (e.g., happier). Compared with the MOA task, the 2IFC task contributes to reduce response time and avoid interference from adjustment stimuli during the response stage (Liberman et al., 2014).

Our second aim (Experiment 2 and 3) was to further investigate the nature of the serial dependence effect of perceived facial expression. Recent work has demonstrated that serial dependence effect is associated with working memory (Papadimitriou et al., 2015; Bliss et al., 2017; Fritsche et al., 2017; Papadimitriou and Snyder, 2017). For example, the serial dependence effect on orientation perception was found to be stronger when the current visual stimulus was retained in working memory for a longer duration in an adjustment task, suggesting that working memory may be a source of this effect (Fritsche, et al., 2017). These findings were in line with previous reports that residual memory for stimuli on previous trials had influence on the judgment of the subsequent trial’s stimuli (Nelson, 1985; Visscher et al., 2009; Huang and Sekuler, 2010). However, whether working memory representations would modulate serial dependence effects of perceived facial expression with a changeable attribute has not been investigated yet.

Collectively, the current study aimed to investigate the serial dependence of perceived emotional expression and its nature. Our results in Experiment 1 showed the existence of the serial dependence of perceived emotional expression. We further found that the serial dependence effect occurred only when the first facial expression was retained in working memory for a longer duration (Experiment 2), and the serial dependence effect became weaker when a longer delay between decision and response was performed (Experiment 3), indicating that working memory maintenance modulated serial dependence of perceived emotional expression.

## Experiment 1: Examining the Serial Dependence of Perceived Emotional Expression Using the Method of Constant Stimuli

In Experiment 1, we aimed to replicate the result of Liberman et al. (2018) experiments where perception of a facial expression was biased toward recently viewed facial expressions (i.e., serial dependence effect of perceived emotional expression), using the method of constant stimuli and a 2IFC task rather than their original MOA task. To verify the reliability of research results, we recruited two groups of participants (Experiment 1a and 1b) separately. They performed the same tasks but did them using different response instructions (see Procedure for details).

### Methods

#### Participants

Sixteen participants (ten females; mean age = 21.2 years, *SD* = 1.9; 19–25 years) participated in Experiment 1a, and an additional sixteen participants (nine females; mean age = 20.8 years, *SD* = 1.4; 19–24 years) participated in Experiment 1b. All participants were naïve to the experiments, except one of the authors (S.C.) who participated in Experiment 1a. All participants of the current study had normal or corrected-normal vision, provided written informed consent and were paid after completing the experiments. All experimental procedures were approved by the School of Psychology Ethics Committee at Guizhou Normal University and conformed to the Declaration of Helsinki.

#### Stimuli and Apparatus

**FIGURE 1:**
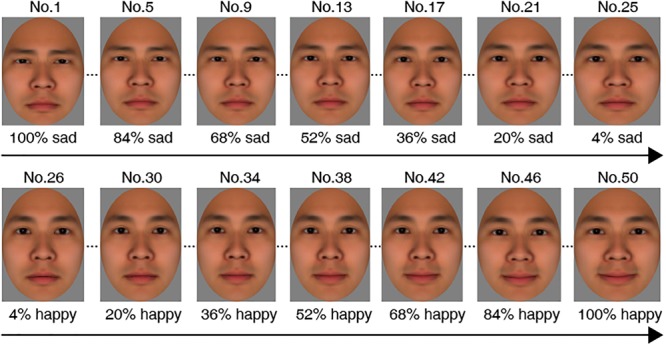
Examples of morphed emotional expressions used in Experiment 1, Experiment 2, and Experiment 3. Fifty morphs with various intensities from 100% sad to 100% happy were created using an Asian male model displaying a neutral expression. For convenience, we coded these morphs from number 1 to 50 along the continuum.

We used FaceGen Modeller 3.4^1^ to generate an Asian male face model which is based on pictures of laser-scanned 3D human faces. FaceGen can generate realistic facial expressions by controlling action units parametrically, based on the Facial Action Coding System. This software has been used to morph various intensities of emotional expressions in previous studies (e.g., Huang et al., 2012; Thoma et al., 2013). In the current study, the “Expression” module of the Modeller was used to morph the FaceGen model with a neutral expression into emotional faces. Using the “Sad Expression” sub-module, we morphed sad expressions with the neutral expression, resulting in a set of 25 sad morphs with various intensities ranging from 4 to 100% sad with 4% increments. Similarly, using the “Happy Expression” sub-module, we morphed happy expressions with the neutral expression, resulting in a set of 25 happy morphs with various intensities ranging from 4 to 100% happy with 4% increments. Thus, a continuum with 50 morphed faces from sad to happy was formed. These morphed faces were coded from number 1 to 50 along the continuum (see Figure 1). All morphed faces were then cropped into oval shapes, using PhotoShop software and presented on a screen with a gray background. Note that the morphing method used in the current study was different than that used by Liberman et al. (2018) in which they generated morphs between two different expressions (e.g., from happy to sad). Morphing sad/happy with neutral could more closely produce natural expressions, compared with morphing between different expressions (Janek and Martin, 2015). The morphing method in the current study was used in previous studies (e.g., Kamachi et al., 2001; Bui et al., 2015; D’Hondt et al., 2015; Jackson and Arlegui-prieto, 2016; Nakajima et al., 2017; Orrie et al., 2018).

Face stimuli (6.1° × 7.9°) were presented on the center of a gamma-corrected 21-in. CRT monitor with a resolution of 1024 × 768 pixels and a refresh rate of 85 Hz. All experiments were programmed using the PsychToolbox-3 (Brainard, 1997; Pelli, 1997) running in MATLAB (MathWorks, Natick, MA, United States). Participants were required to view face stimuli at a distance of 56 cm in a dark room, and their heads were rested on a chinrest. The same facial expression stimuli and apparatus were used for all experiments.

#### Procedure

**FIGURE 2:**
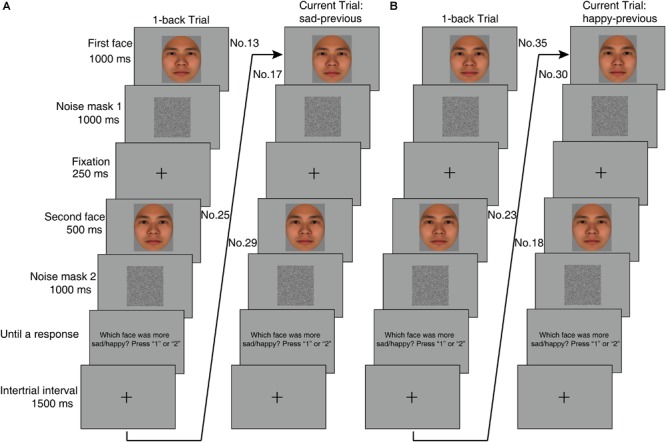
The schematic description of the trial sequences for the two-interval forced choice (2IFC) task and trial types in Experiment 1. The sad-previous trial **(A)** and happy-previous trial **(B)** were displayed separately. In each trial, the first facial expression was presented for 1000 ms, followed by a 1000 ms noise mask and a 250 ms fixation cross. Then the second facial expression was presented for 500 ms, and after a 1000 ms noise mask, the question “which facial expression was more sad” (Experiment 1a) or “which facial expression was more happy” (Experiment 1b) appeared until participants made a response by pressing “1” or “2” on the keypad. The next trial started after a 1500 ms intertrial interval. We classified the trial types based upon the position of the n-back first emotional expression in the morph continuum (see Analysis for details).

The method of constant stimuli and the 2IFC task was used. In each trial (see Figure 2), two facial expressions were presented one after another. The first facial expression was presented for 1000 ms, followed by a 1000 ms noise mask with a random level of intensity in grayscale for each pixel and then a 250 ms fixation cross. Next, the second facial expression was presented for 500 ms, and after a 1000 ms noise mask, the question “which facial expression was more sad” (Experiment 1a) or “which facial expression was more happy” (Experiment 1b) appeared until participants made a response by pressing “1” or “2” on the keypad. The next trial started after a 1500 ms intertrial interval. The participants were allowed to guess if necessary.

In regard to different exposure durations of the first and the second facial expressions, we followed the trial sequence for the 2IFC task in Experiment 2 of Liberman et al. (2014), although they investigated the serial dependence effect in the perception of face identity rather than emotional expressions. The same rationale and parameters for exposure durations as that in experiment 2 of Liberman et al. (2014) were used. We hypothesized that the one-back first facial expression had a stronger pull to the next trial’s first facial expression than the one-back second facial expression, given that the exposure duration of the first facial expression (i.e., 1000 ms) doubles as that of the second facial expression (i.e., 500 ms). If equal exposure durations for the first and the second facial expressions were used in a 2IFC task, the one-back serial dependence effect could not be found. This is because the first and the second facial expressions may have different emotional dimensions (i.e., sad versus happy), thereby producing opposite equal pulls to the next trial’s first facial expression. These opposite pulls may cancel each other out, leading to no serial dependence effect.

Following the previous work (Liberman et al., 2014), we randomly selected the first facial expression from 26 morphed expressions ranging from number 13 to 38 in the center of the morphed continuum in each trial. The second facial expression could have differences of ±12, ±6, or 0 bins from the first facial expression. For example, the facial expression numbered 1 should be selected as the second facial expression toward the -12 bin when a facial expression numbered 13 was selected as the first facial expression. The second facial expression displayed a more sad expression (or less happy) than the first facial expression when -12 and -6 bins were used; the second facial expression displayed less sad (or a more happy expression) than the first facial expression when +12 and +6 bins were used. The same first and second facial expressions were used in the 0 bin.

Because 26 morphed expressions for the first facial expression and five types of bins for the second facial expression were available in each trial, 130 trials were included in each session. Each participant finished three sessions (i.e., 390 trials) in total and had a break for at least 10 min between two successive sessions. Each session took approximately 13 min. Participants completed 20 practice trials in which feedback was provided but not in the formal experiments.

#### Analysis

According to previous studies (Liberman et al., 2014; Taubert et al., 2016a) and our current design, we classified trials into two subgroups: “sad-previous” trials and “happy-previous” trials. The former referred to those trials for which the one-back first facial expression belonged to the part including sad morphs (i.e., number 13 to 25) in the continuum; the latter referred to those trials for which the one-back first facial expression belonged to the part including happy morphs (i.e., number 26 to 38) in the continuum. Participants finished equal numbers of “sad-previous” trials and “happy-previous” trials. These two types of trials were randomly presented. For each participant, psychometric functions of the “sad-previous” trials and the “happy-previous” trials were separately fitted with a logistic equation as follows:

$$\begin{array}{c} P=\frac{1}{1+e^{-a\left(x-b\right)}} \end{array}$$

Where x represents five bins (i.e., ± 12, ± 6 and 0), P represents percent of first faces chosen as more sad or happy on each bin, the parameters a and b are the slope and the point of subjective equality (PSE) for the logistic function, respectively. For each participant, the difference of the PSE values (ΔPSE) between the “sad-previous” trials and the “happy-previous” trials was calculated. This difference would be zero if serial dependence or visual adaptation failed to occur. Using one-sample *t*-test, we evaluated statistical significance between PSE differences and zero. For the *t*-tests, the Cohen’s *d* was used as an indicator for the effect size (Cohen, 1992).

### Results

**FIGURE 3:**
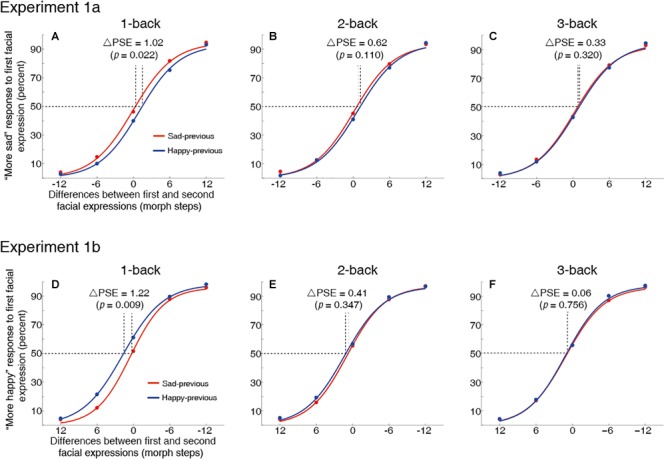
Grand average psychometric functions for Experiment 1a (top panel) and 1b (bottom panel). The horizontal coordinate represents the differences between the first and second facial expressions in the morph continuum. Trials in the –6 and –12 bin denote that the second facial expression displayed more sad (or less happy) than the first facial expression; trials in the 6 and 12 bin denote that the second facial expression displayed less sad (or more happy) than the first facial expression; trials in the 0 bin denote the same first and second facial expressions. Note that, for comparison, Experiment 1a and 1b have an opposite direction on the horizontal coordinate values. The vertical coordinate represents percent of the first facial expression chosen as more sad (Experiment 1a) or more happy (Experiment 1b) on the corresponding five bins. The red and blue curves represent the fitting curves for the “sad-previous” and “happy-previous” trials, respectively. ΔPSE denotes the differences of in PSE values (i.e., magnitude of serial dependence effects) between the “sad-previous” trials and the “happy-previous” trials. One-back, two-back and three-back serial dependence effects are shown separately in the top panel **(A–C)** for Experiment 1a and in the bottom panel **(D–F)** for Experiment 1b. See Procedure and Analysis for more details.

In Experiment 1a, a significant leftward shift of the one-back sad-previous curve relative to the 1-back happy-previous curve (i.e., significant ΔPSE) was found [*t*(15) = 2.55, *p* = 0.022, *d* = 0.64] (Figure 3A); similarly, in Experiment 1b a significant leftward shift of the one-back happy-previous curve relative to the one-back sad-previous was also found [*t*(15) = 2.98, *p* = 0.009, *d* = 0.75] (Figure 3D). These results suggested that perception of the current trial’s first facial expression was pulled toward one-back trial’s first facial expression (one-back serial dependence effect).

There were no 2-back serial dependence effect [Experiment 1a: *t*(15) = 1.70, *p* = 0.110, *d* = 0.43, see Figure 3B; Experiment 1b: *t*(15) = 0.97, *p* = 0.347, *d* = 0.24, see Figure 3E] and 3-back serial dependence effects [Experiment 1a: *t*(15) = 1.03, *p* = 0.320, *d* = 0.26, see Figure 3C; Experiment 1b: *t*(15) = 0.32, *p* = 0.756, *d* = 0.08, see Figure 3F]. Similar results of Experiment 1a and 1b indicated that different response instructions had no impact on the serial dependence effect in the perception of perceived facial expression.

Given that average response times across participants were about 604 ± 228 ms in Experiment 1a and about 512 ± 154 ms in Experiment 1b, the one-back first facial expression appeared before the current first facial expression about 5854 ms and 5762 ms before in Experiment 1a and 1b, respectively. In addition, the fitted slopes (i.e., the parameter *a*) between the “sad-previous” and the “happy-previous” curves showed no significant difference [Experiment 1a: *t*(15) = 0.92, *p* = 0.374, *d* = 0.23; Experiment 1b: *t*(15) = 1.29, *p* = 0.216, *d* = 0.32], indicating that there was no difference in sensitivity between these two types of trials. Similar results were found when the author’s (S.C.) data was removed for Experiment 1a (see Supplementary Materials for details).

## Experiment 2: Stronger Serial Dependence of Perceived Emotional Expression When Lengthening the Duration of the First Face Retained in Working Memory

Experiment 1 replicated and extended previous results demonstrating the existence of the serial dependence of perceived emotional expression (Liberman et al., 2018), using a different method (constant stimuli versus adjustment). Although recent works have demonstrated that working memory representations are associated with the serial dependence effects of stable attributes of visual stimuli (Papadimitriou et al., 2015; Bliss et al., 2017; Fritsche et al., 2017; Papadimitriou and Snyder, 2017), it is unclear whether the serial dependence effect of perceived emotional expression (a changeable visual attribute) would be modulated by working memory representation.

Given that the design of Experiment 1 required participants to compare the first faces with the second faces across time in the 2IFC task, a working memory component was necessarily involved. More specifically, in a 2IFC task participants need to judge how a stimulus they are looking at differs from another stimulus presented a few seconds ago, so they must draw on their memory for making the judgment. When the time interval between the two stimuli is longer, serial dependence from previous trials may impact upon their judgment more. In Experiment 2, we shortened or lengthened the duration of the first face retained in working memory in a 2IFC task to observe whether serial dependence effects would emerge.

### Methods

#### Participants

Fifteen participants (eight females; mean age = 20.3 years, *SD* = 1.1; 19–22 years) participated in Experiment 2. Except for one of the authors (S.C.), all participants were naïve to the experiment. One additional participant was excluded because her data were greater than two standard deviations.

#### Stimuli, Apparatus, Procedure, and Analysis

**FIGURE 4:**
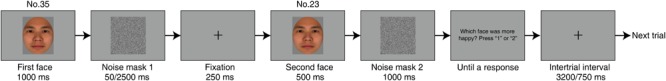
The trial procedure of Experiment 2. In each trial, the first facial expression was presented for 1000 ms, followed by a 50 ms or a 2500 ms noise mask 1. These two various durations were counterbalanced across sessions. After a 250 ms fixation cross, the second facial expression was presented for 500 ms, followed by a 1000 ms noise mask 2. Then the question “which facial expression was more happy” appeared until participants made a response by pressing “1” or “2.” The next trial started after a 3200 ms or a 750 ms intertrial interval, depending on the corresponding delay durations of noise mask 1.

Stimuli, apparatus, procedure, and analysis were the same as Experiment 1, except for the following trial sequence (see Figure 4). In Experiment 2, we used various time intervals between the first and the second faces by shortening the duration (50 ms) or lengthening the duration (2500 ms) of the noise mask 1. This design can lead to different durations for which the first emotional expression was retained in working memory before giving a final response for participants. The 50 ms and 2500 ms duration conditions were counterbalanced across sessions. All participants finished three sessions for each condition. In addition, to ensure an approximate equal time interval between the first face of the current trial and the next trial under the two delay duration conditions, we used different intertrial intervals (3200 ms or 750 ms) for the corresponding delay durations. Participants were instructed to decide which facial expression was perceived as happier.

### Results

**FIGURE 5:**
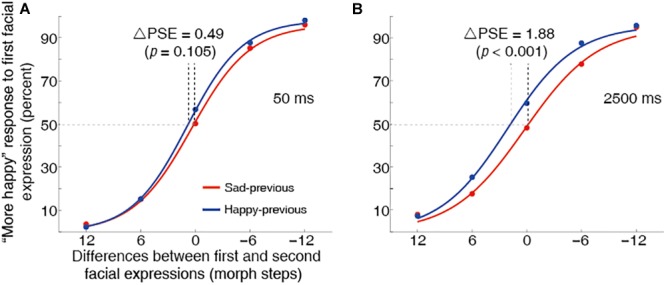
Grand average psychometric functions for the 50 ms **(A)** and 2500 ms **(B)** delay duration conditions in Experiment 2. Plotting conventions are same as in Figure 3. The one-back serial dependence effect occurred in the 2500 ms delay duration condition but did not in the 50 ms delay duration condition.

A significant leftward shift of the happy-previous curve relative to the sad-previous was found for the 2500 ms delay duration condition [*t*(13) = 5.08, *p* < 0.001, *d* = 1.36] (Figure 5B), suggesting that a one-back serial dependence effect of the perceived emotion expression occurred. This result was consistent with the findings of Experiment 1. However, for the 50 ms delay duration condition, no serial dependence effect was observed [*t*(13) = 1.74, *p* = 0.104, *d* = 0.47] (Figure 5A). Thus, working memory representation may modulate the serial dependence effect of a perceived emotional expression. Similar results were found when the author’s (S.C.) data was removed for Experiment 2 (see Supplementary Materials for details).

## Experiment 3: Weaker Serial Dependence of Perceived Emotional Expression When Lengthening the Time Interval Between Decision and Response

In Experiment 2, we found the serial dependence effect only when the first faces had to be retained in working memory for longer periods in a 2IFC task. In other words, maintaining the current first face in working memory for a longer period caused it to be recalled as more similar to the one-back first face. In Experiment 3 we further investigated whether memory decay would attenuate the serial dependence effect when a longer delay between decision and response was performed. In a 2IFC task, participants could make their decision as soon as the second face was presented, and they would only need to remember their decision until the response period started. Thus, more attention could be allocated to decision results rather than the current stimuli *per se* when a longer delay between decision and the response period was performed. Given that working memory and attention are considered to share the overlapping mechanisms (for reviews, Awh and Jonides, 2001; Awh et al., 2006; Gazzaley and Nobre, 2012; Kiyonaga and Egner, 2013; Myers et al., 2017), we predicted that a weaker serial dependence effect would be observed in the longer-delay condition.

### Methods

#### Participants

Seventeen participants (13 females; mean age = 20.3 years, *SD* = 1.0; 19–22 years) participated in Experiment 3. Four of the participants in Experiment 3 had participated in Experiment 1b. Except for one of the authors (S.C.), all participants were naïve with respect to the purpose of the experiment. Two additional participants were excluded because one did not follow the instructions and one’s data were greater than the two standard deviations.

#### Stimuli, Apparatus, Procedure, and Analysis

**FIGURE 6:**
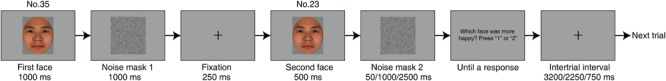
The trial procedure of Experiment 3. In each trial, the first facial expression was presented for 1000 ms, followed by a 1000 ms noise mask 1. After a 250 ms fixation cross, the second facial expression was presented for 500 ms, followed by a 50 ms, a 1000 ms, or a 2500 ms noise mask 2. These three various durations were counterbalanced across sessions. Then the question “which facial expression was happier” appeared until participants made a response by pressing “1” or “2.” The next trial started after a 3200 ms, a 2250 ms, or a 750 ms intertrial interval, depending on the corresponding delay durations of noise mask 2.

Stimuli, apparatus, procedure, and analysis were the same as in Experiment 2 except for following (see Figure 6). In Experiment 3, we used various time intervals between the second face and the response introduction, using the three durations of the noise mask 2 (50 ms, 1000 ms and 2500 ms). The second faces would be retained in working memory for a longer period when a longer duration of the noise mask 2 was used. The three delay duration conditions were counterbalanced across sessions. All participants finished three sessions for each condition. Intertrial intervals included 3200, 2250, and 750 ms, depending on the delay duration of the noise mask 2. In addition, a One-Way Repeated Measures ANOVA was performed to compare the differences on the magnitude of the serial dependence effects for the 50, 1000, and 2500 ms delay duration conditions. For the ANOVA test, partial eta-squared ($\eta_{p}^{2}$) was used as an estimate of the effect size (Cohen, 1973).

### Results

**FIGURE 7:**
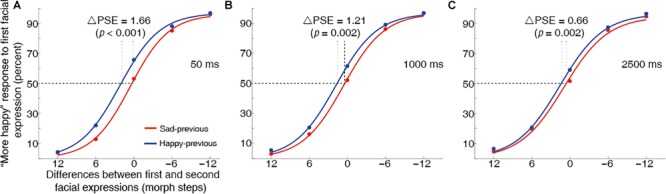
Grand average psychometric functions for the 50 ms **(A)**, 1000 ms **(B)** and 2500 ms **(C)** delay duration conditions in Experiment 3. Plotting conventions are the same as in Figure 3. The one-back serial dependence effect was significantly stronger in the 50 ms delay duration condition than that in the 2500 ms delay duration condition.

For all three delay duration conditions, significant leftward shifts of happy-previous curve relative to the sad-previous were observed [50 ms: *t*(16) = 5.64, *p* < 0.001, *d* = 1.37; 1000 ms: *t*(16) = 3.75, *p* = 0.002, *d* = 0.91; 2500 ms: *t*(16) = 3.67, *p* = 0.002, *d* = 0.89], demonstrating one-back serial dependence effects (Figure 7). More importantly, a One-Way Repeated Measures ANOVA suggested that the time interval between decision and the response period had a significant effect on the magnitude of the serial dependence effects (i.e., ΔPSE) [*F*(_2,32_) = 3.42, *p* = 0.045, $\eta_{p}^{2}$ = 0.18]. As expected, the *post hoc* paired comparisons demonstrated that the magnitude of the serial dependence effect of the 50 ms delay duration condition was significantly greater than that of the 2500 ms delay duration condition (*p* = 0.007), suggesting that lengthening the time interval between decision and the response period reduced the serial dependence effect toward a one-back first emotional expression, due to memory decay. Thus, the findings of Experiment 3 were consistent with that of Experiment 2, indicating that working memory maintenance modulated the serial dependence effect of perceived emotional expression. In addition, although an inspection of Figure 5 found that the magnitude of the serial dependence effects decreased as the time interval between decision and the response period increased, the differences between the 50 ms and 1000 ms delay duration conditions and between the 1000 ms and 2500 ms delay duration conditions did not reach a statistical significance (*p* = 0.296 and *p* = 0.180). Similar results were found when the author’s (S.C.) data was removed for Experiment 3 (see Supplementary Materials for details).

## Discussion

Using three psychophysical experiments, we revealed the serial dependence effect of perceived emotional expression and its dependence on working memory. Experiment 1a and 1b demonstrated that perceived emotional expression was pulled by the one-back expression occurring on the ∼6 s before, and this positive serial dependence effect was independent of response instructions. Experiment 2 suggested that the serial dependence effect of perceived emotional expression occurred only when working memory representation toward the first emotional expression was retained for a longer duration. Experiment 3 further showed that the serial dependence effect was weakened when a longer delay between decision and response was performed. These results attest the existence of serial dependence in facial emotional expression and indicate that working memory representations modulate the serial dependence effect of perceived emotional expression.

In the current study, we replicated Liberman et al.’s (2018) findings about the serial dependence effect in perceived emotional expression. The 2IFC task was used in our work, rather than the MOA task used in Liberman et al.’s (2018) work, indicating that the serial dependence in perceived emotional expression did not depend on experimental tasks. However, our current results were inconsistent with Taubert et al.’s (2016a) study in which the serial dependence effect of perceived facial emotion expression failed to be observed. One of the possible reasons is that in their study facial emotional expressions presented on the current and previous trial involved different genders of faces. As shown in Experiment 2 of Liberman et al. (2018), a pull in perceived emotional expression toward recently viewed expression occurred only under a condition in which the current and previous emotional expression had the same gender. In our experiments the same gender was also used for the current and previous trial. Thus, the serial dependence of perceived emotional expression indeed exists but may depend on face similarity.

Previous studies have demonstrated clear positive serial dependencies in the perception of stable attributes of faces (e.g., Liberman et al., 2014; Taubert et al., 2016a; Kok et al., 2017). Our current study and Liberman et al.’s (2018) work both show that even for the perception of changeable attributes like facial emotional expression, there are serial dependence effects. This mechanism promotes us to perceive an individual’s emotional expression as stable in social interactions during a certain period. After all, it is impossible that emotional expressions of an individual would change from moment to moment despite being less stable relative to other attributes of faces like gender. Recent work showed that another changeable attribute of faces – eye gaze direction – also exhibited the serial dependence effect (Alais et al., 2018). More research should be carried out to investigate whether serial dependence effects exist for other changeable attributes of faces such as face viewpoint. In addition, to maintain high sensitivity to detect new expressions, the visual system simultaneously also exhibits visual adaptation (i.e., negative aftereffects) in the perception of emotional expression (Webster et al., 2004; Fox and Barton, 2007; Ellamil et al., 2008; Campbell and Burke, 2009; Ying and Xu, 2017; Palermo et al., 2018). Serial dependence and visual adaptation together shape our perception of emotional expressions. How these two mechanisms interact in the perception of emotional expression remains an open question.

Experiment 2 showed that the serial dependence effect was magnified when the first face was in the working memory for longer, consistent with previous results obtained by using the MOA task (Bliss et al., 2017; Fritsche et al., 2017). In the MOA task, to perform an adjustment response, participants need to remember the target stimulus. The longer the time interval between the target stimulus and adjustment stimulus is, the longer the target stimulus is retained in working memory. Similarly, in the 2IFC task, to perform the comparison of two target faces in the response period, participants need to remember the two target faces. The longer the time interval between the first and the second target faces is, the longer the first face is retained in working memory. Thus, as Experiment 2 suggested, a stronger serial dependence effect of perceived emotional expression appeared when the time interval became longer between the first and the second faces. Furthermore, in Experiment 3 we controlled the time interval between the second face and the start of the responding period and predicted that memory decay would happen in the longer-decay condition. This would attenuate the serial dependence effect. As expected, the results of Experiment 3 attested the hypothesis. Therefore, Experiment 2 and 3 both provided evidence that working memory representation modulated the serial dependence effect in the perception of emotional expression. Previous studies have examined the role of working memory toward the serial dependence effects of the basic visual features such as orientation (Fritsche et al., 2017) and position (Papadimitriou et al., 2015; Bliss et al., 2017; Papadimitriou and Snyder, 2017). Our current results demonstrates that working memory also modulates the serial dependence of the high-level visual attributes such as emotional expressions.

Furthermore, taken together with past work (Bliss et al., 2017; Fritsche et al., 2017), the results of Experiment 2 indicated that working memory may help consolidate memory representations toward the recently seen stimuli over time and thus increases the magnitude of the serial dependence effect. A previous study suggested that visual short-term maintenance also increased the magnitude of the tilt negative aftereffect (Saad and Silvanto, 2013). These results indicate that memory consolidation works not only in visual adaptation but also in serial dependence. However, memory consolidation can be active in serial dependence only when memory demands are prolonged (e.g., in Experiment 2 working memory representations of first faces must be retained to perform the comparison of two faces presented sequentially). As the results of Experiment 3 suggested, memory decay rather than memory consolidation appeared when the face stimuli was not active in working memory, resulting in the attenuation of the magnitude of the serial dependence effect. In line with this view, recently Bliss et al. (2017) found that the magnitude of the serial dependence effect decreased with the mere passage of time. The time course of the serial dependence effect in working memory needs to be further investigated in future work.

However, an alternative explanation was possible with regards to the result of Experiment 2 in which no serial dependence effect was observed for the 50 ms delay duration condition. Because positive aftereffects (i.e., serial dependence) and negative aftereffects (i.e., visual adaptation) may be present at the same time in visual perception (Fischer and Whitney, 2014; Taubert et al., 2016a; Alais et al., 2017; Cicchini et al., 2017; Manassi et al., 2018), both these opposing biases could cancel each other out for the 50 ms delay duration condition. In future work, the role of mask duration in the perception of perceived emotional expression should be explored.

In the current study, the serial dependence effects (ΔPSE) ranging from 1.02 to 1.88 morph steps seems small. Previous studies using a similar task can provide a reference for the effect size. Given that the current study followed the design of Liberman et al. (2014) in Experiment 2, similar 2IFC task and stimulus parameters (e.g., ±12, and ±6 morph step differences for the two faces) were used. In fact, in their study the serial dependence effect (ΔPSE) also seemed small (i.e., about 2 morph steps). We imagine that the reported effects, which were smaller than morph steps (i.e., six steps) used as differences in constant stimuli in the 2IFC task, are associated with the selection of stimulus levels when using the method of constant stimuli and the 2IFC task. To efficiently fit the psychometric function and obtain PSE when using the method of constant stimuli, the minimum and maximum stimulus levels need to be included and various stimulus levels need to be as differentiable as possible. Therefore, as previous studies suggest, the estimated PSE is usually smaller than the differences in stimulus levels that are used as constant stimuli in serial dependence (Liberman et al., 2014; Fritsche et al., 2017). In addition, some other factors may contribute to the strength of the serial dependence effects such as the stimulus duration, the time interval between adjacent trials, the magnitude of the stimuli (e.g., use the most extreme emotional expressions for the first face). In particular, because the maximum difference for the first faces of adjacent trials was 25 morph bins in the current study, the size of the serial dependence effect could be reduced. Therefore, which factors influenced the strength of the serial dependence effect should be systematically investigated in future work.

In summary, using psychophysical experiments, we observed the serial dependence effect of the perceived emotional expression, and found that working memory modulated this effect. These results, taken together with previous works, suggest that the Continuity Field appears to operate on not only stable (e.g., face identity) but also on changeable (e.g., emotional expression) attributes of faces.

## Data Availability

All datasets generated for this study are included in the manuscript and/or the Supplementary Files.

## Ethics Statement

All participants of the current study had normal or corrected-normal vision, gave their written informed consent and were paid after completing the experiments. All experimental procedures were approved by the School of Psychology Ethics Committee at Guizhou Normal University, and conformed to the Declaration of Helsinki.

## Author Contributions

GM designed the research. SC performed the research. GM and SC analyzed the data. GM, SC, and BD wrote the manuscript.

## Conflict of Interest Statement

The authors declare that the research was conducted in the absence of any commercial or financial relationships that could be construed as a potential conflict of interest.

## Footnotes

1 http://www.facegen.com/

## References

[B1] Alais D. Kong G. Palmer C. Clifford C. (2018). Eye gaze direction shows a positive serial dependency. *J. Vision* 18:11. 10.1167/18.4.11 29710301

[B2] Alais D. Leung J. Van D. Burg E. (2017). Linear summation of repulsive and attractive serial dependencies: orientation and motion dependencies sum in motion perception. *J. Neurosci.* 37 4601–4615. 10.1523/JNEUROSCI.4601-15.2017 28330878 PMC6596560

[B3] Alexi J. Cleary D. Dommisse K. Palermo R. Kloth N. Burr D. (2018). Past visual experiences weigh in on body size estimation. *Sci. Rep.* 8:215. 10.1038/s41598-017-18418-3 29317693 PMC5760712

[B4] Awh E. Jonides J. (2001). Overlapping mechanisms of attention and spatial working memory. *Trends Cogn. Sci.* 5 119–126. 10.1016/s1364-6613(00)01593-x 11239812

[B5] Awh E. Vogel E. K. Oh S. H. (2006). Interactions between attention and working memory. *Neuroscience* 139 201–208. 10.1016/j.neuroscience.2005.08.023 16324792

[B6] Bliss D. P. Sun J. J. D’Esposito M. (2017). Serial dependence is absent at the time of perception but increases in visual working memory. *Sci. Rep.* 7:14739. 10.1038/s41598-017-15199-7 29116132 PMC5677003

[B7] Brainard D. H. (1997). The psychophysics toolbox. *Spat. Vision* 10 433–436. 10.1163/156856897x00357 9176952

[B8] Bui E. Anderson E. Goetter E. M. Campbell A. A. Fischer L. E. Barrett L. F. (2015). Heightened sensitivity to emotional expressions in generalised anxiety disorder, compared to social anxiety disorder, and controls. *Cogn. Emot.* 31 119–126. 10.1080/02699931.2015.1087973 26395075 PMC5199214

[B9] Campbell J. Burke D. (2009). Evidence that identity-dependent and identity-independent neural populations are recruited in the perception of five basic emotional facial expressions. *Vision Res.* 49 1532–1540. 10.1016/j.visres.2009.03.009 19303422

[B10] Cicchini G. M. Anobile G. Burr D. C. (2014). Compressive mapping of number to space reflects dynamic encoding mechanisms, not static logarithmic transform. *Proc. Natl. Acad. Sci. U.S.A.* 111 7867–7872. 10.1073/pnas.1402785111 24821771 PMC4040572

[B11] Cicchini G. M. Mikellidou K. Burr D. (2017). Serial dependencies act directly onperception. *J. Vision* 17 1–9. 10.1167/17.14.6 29209696

[B12] Cohen J. (1973). Eta-squared and partial eta-squared in fixed factor anova designs. *Educ. Psychol. Measure.* 33 107–112. 10.1177/001316447303300111

[B13] Cohen J. (1992). A power primer. *Tutor. Q. Methods Psychol.* 3 155–159. 10.1037//0033-2909.112.1.155 19565683

[B14] Corbett J. E. Fischer J. Whitney D. (2011). Facilitating stable representations: serial dependence in vision. *PLoS One* 6:e16701. 10.1371/journal.pone.0016701 21304953 PMC3031612

[B15] D’Hondt F. de Timary P. Bruneau Y. Maurage P. (2015). Categorical perception of emotional facial expressions in alcohol-dependence. *Drug Alcohol Depend.* 156 267–274. 10.1016/j.drugalcdep.2015.09.017 26433563

[B16] Ellamil M. Susskind J. M. Anderson A. K. (2008). Examinations of identity invariance in facial expression adaptation. *Cogn. Affect. Behav. Neurosci.* 8 273–281. 10.3758/cabn.8.3.273 18814464

[B17] Fischer J. Whitney D. (2014). Serial dependence in visual perception. *Nat. Neurosci.* 17 738–743. 10.1038/nn.3689 24686785 PMC4012025

[B18] Fornaciai M. Park J. (2018). Attractive serial dependence in the absence of an explicit task. *Psychol. Sci.* 29 437-446. 10.1177/0956797617737385 29381415

[B19] Fox C. J. Barton J. J. (2007). What is adapted in face adaptation? the neural representations of expression in the human visual system. *Brain Res.* 1127 80–89. 10.1016/j.brainres.2006.09.104 17109830

[B20] Fritsche M. Mostert P. de Lange F. P. (2017). Opposite effects of recent history on perception and decision. *Curr. Biol.* 27 590–595. 10.1016/j.cub.2017.01.006 28162897

[B21] Gazzaley A. Nobre A. C. (2012). Top-down modulation: bridging selective attention and working memory. *Trends Cogn. Sci.* 16 129–135. 10.1016/j.tics.2011.11.014 22209601 PMC3510782

[B22] Hsu S. M. Young A. (2004). Adaptation effects in facial expression recognition. *Visual Cogn.* 11 871–899. 10.1080/13506280444000030

[B23] Huang J. Sekuler R. (2010). Distortions in recall from visual memory: two classes of attractors at work. *J. Vision* 10 1–27. 10.1167/10.2.24 20462325 PMC4104522

[B24] Huang L. C. Hsiao S. Hwu H. G. Howng S. L. (2012). The chinese facial emotion recognition database (cferd): a computer-generated 3-d paradigm to measure the recognition of facial emotional expressions at different intensities. *Psychiatry Res.* 200 928–932. 10.1016/j.psychres.2012.03.038 22503384

[B25] Jackson M. C. Arlegui-prieto M. (2016). Variation in normal mood state influences sensitivity to dynamic changes in emotional expression. *Emotion* 16 145–149. 10.1037/emo0000126 26479773

[B26] Janek L. Martin F. (2015). Facial feedback affects perceived intensity but not quality of emotional expressions. *Brain Sci.* 5 357–368. 10.3390/brainsci5030357 26343732 PMC4588143

[B27] Kamachi M. Bruce V. Mukaida S. Gyoba J. Yoshikawa S. Akamatsu S. (2001). Dynamic properties influence the perception of facial expressions. *Perception* 30 875–887. 10.1068/p3131 11515959

[B28] Kiyonaga A. Egner T. (2013). Working memory as internal attention: toward an integrative account of internal and external selection processes. *Psychon. Bull. Rev.* 20 228–242. 10.3758/s13423-012-0359-y 23233157 PMC3594067

[B29] Kiyonaga A. Scimeca J. M. Bliss D. P. Whitney D. (2017). Serial dependence across perception, attention, and memory. *Trends Cogn. Sci.* 21 493–497. 10.1016/j.tics.2017.04.011 28549826 PMC5516910

[B30] Kohn A. (2007). Visual adaptation: physiology, mechanisms, and functional benefits. *J. Neurophysiol.* 97 3155–3164. 10.1152/jn.00086.2007 17344377

[B31] Kok R. Taubert J. Van D. Burg E. Rhodes G. Alais D. (2017). Face familiarity promotes stable identity recognition: exploring face perception using serial dependence. *R. Soc. Open Sci.* 4:160685. 10.1098/rsos.160685 28405355 PMC5383812

[B32] Liberman A. Fischer J. Whitney D. (2014). Serial dependence in the perception of faces. *Curr. Biol.* 24 2569–2574. 10.1016/j.cub.2014.09.025 25283781 PMC4254333

[B33] Liberman A. Manassi M. Whitney D. (2018). Serial dependence promotes the stability of perceived emotional expression depending on face similarity. *Attent. Percep. Psychophy.* 80 1461–1473. 10.3758/s13414-018-1533-8 29736808

[B34] Liberman A. Zhang K. Whitney D. (2016). Serial dependence promotes object stability during occlusion. *J. Vision* 16 1–10. 10.1167/16.15.16 PMC5214222 28006066

[B35] Manassi M. Liberman A. Chaney W. Whitney D. (2017). The perceived stability of scenes: serial dependence in ensemble representations. *Sci. Rep.* 7:1971. 10.1038/s41598-017-02201-5 28512359 PMC5434007

[B36] Manassi M. Liberman A. Kosovicheva A. Zhang K. Whitney D. (2018). Serial dependence in position occurs at the time of perception. *Psychon. Bull. Rev.* 16 1–9. 10.3758/s13423-018-1454-5 29582377

[B37] Myers N. E. Stokes M. G. Nobre A. C. (2017). Prioritizing information during working memory: beyond sustained internal attention. *Trends Cogn. Sci.* 21 449–461. 10.1016/j.tics.2017.03.010 28454719 PMC7220802

[B38] Nakajima K. Minami T. Nakauchi S. (2017). Interaction between facial expression and color. *Sci. Rep.* 7:41019. 10.1038/srep41019 PMC5259783 28117349

[B39] Nelson T. O. (1985). Ebbinghaus’s contribution to the measurement of retention: savings during relearning. *J. Exp. Psychol. Learn. Memory Cogn.* 11 472–479. 10.1037/0278-7393.11.3.472 3160812

[B40] Orrie D. Iris H. Kfir A. Kesem N. Ami C. (2018). The effect of sleep deprivation on recognition of ambiguous emotional facial expressions in individuals with ADHD. *J. Attent. Disord.* 10.1177/1087054718785473 [Epub ahead of print]. 29973106

[B41] Palermo R. Jeffery L. Lewandowsky J. Fiorentini C. Irons J. L. Dawel A. (2018). Adaptive face coding contributes to individual differences in facial expression recognition independently of affective factors. *J. Exp. Psychol. Hum. Percep. Perform.* 44 503–517. 10.1037/xhp0000463 28825500

[B42] Papadimitriou C. Ferdoash A. Snyder L. H. (2015). Ghosts in the machine: memory interference from the previous trial. *J. Neurophysiol.* 113 567–577. 10.1152/jn.00402.2014 25376781 PMC4297789

[B43] Papadimitriou C. Snyder L. H. (2017). Ghosts in the machine ii: neural correlates of memory interference from the previous trial. *Cereb. Cortex* 27:bhw106. 10.1093/cercor/bhw106 27114176 PMC6059123

[B44] Pelli D. G. (1997). The VideoToolbox software for visual psychophysics: transforming numbers into movies. *Spatial Vision* 10 437–442. 10.1163/156856897x00366 9176953

[B45] Saad E. Silvanto J. (2013). How visual short-term memory maintenance modulates subsequent visual aftereffects. *Psychol. Sci.* 24 803–808. 10.1177/0956797612462140 23558546

[B46] Taubert J. Alais D. Burr D. (2016a). Different coding strategies for the perception of stable and changeable facial attributes. *Sci. Rep.* 6:32239. 10.1038/srep32239 27582115 PMC5007489

[B47] Taubert J. Burg E. V. D. Alais D. (2016b). Love at second sight: sequential dependence of facial attractiveness in an on-line dating paradigm. *Sci. Rep.* 6:22740. 10.1038/srep22740 26986828 PMC4795074

[B48] Thoma P. Soria Bauser D. Suchan B. (2013). Besst (bochum emotional stimulus set)-a pilot validation study of a stimulus set containing emotional bodies and faces from frontal and averted views. *Psychiatry Res.* 209 98–109. 10.1016/j.psychres.2012.11.012 23219103

[B49] Visscher K. M. Kahana M. J. Sekuler R. (2009). Trial-to-trial carryover in auditory short-term memory. *J. Exp. Psychol. Learn. Memory Cogn.* 35 46–56. 10.1037/a0013412 19210080 PMC2744086

[B50] Webster M. A. (2011). Adaptation and visual coding. *J. Vision* 11 74–76. 10.1167/11.5.3 PMC3245980 21602298

[B51] Webster M. A. (2015). Visual adaptation. *Ann. Rev. Vision Sci.* 1 547–567. 10.1146/annurev-vision-082114-035509 PMC4742349 26858985

[B52] Webster M. A. Kaping D. Mizokami Y. Duhamel P. (2004). Adaptation to natural facial categories. *Nature* 428 557–561. 10.1038/nature02420 15058304

[B53] Xia Y. Leib A. Y. Whitney D. (2016). Serial dependence in the perception of attractiveness. *J. Vision* 28 1–8. 10.1167/16.15.28 28006077 PMC5214899

[B54] Ying H. Xu H. (2017). Adaptation reveals that facial expression averaging occurs during rapid serial presentation. *J. Vision* 17 1–19. 10.1167/17.1.15 28114490

