# Supplementary material for: Working Memory Maintenance Modulates Serial Dependence Effects of Perceived Emotional Expression
Source: Front Psychol. 2019 Jul 11;10:1610. doi: 10.3389/fpsyg.2019.01610 (PMC6637952; doi:10.3389/fpsyg.2019.01610)
Supplement: Supplementary file 1 [file Table_1.DOCX]

**Supplementary Materials**

Gaoxing Mei, Shiyu Chen, Bo Dong

The results of Experiment 1a, Experiment 2 and Experiment 3 were re-analyzed after the author (S.C.) 'data was removed, as follows.

**Experiment 1a**

A significant leftward shift of the one-back sad-previous curve relative to the 1-back happy-previous curve (i.e. significant △PSE) was found (*t*(14) = 2.20, *p* = 0.046, *d* = 0.57) (Figure S1(A)). There were no a 2-back serial dependence effect (*t*(14) = 1.57, *p* = 0.140, *d* = 0.40, see Figure S1(B)) and a 3-back serial dependence effect (*t*(14) = 0.74, *p* = 0.469, *d* = 0.19, see Figure S1(C)).


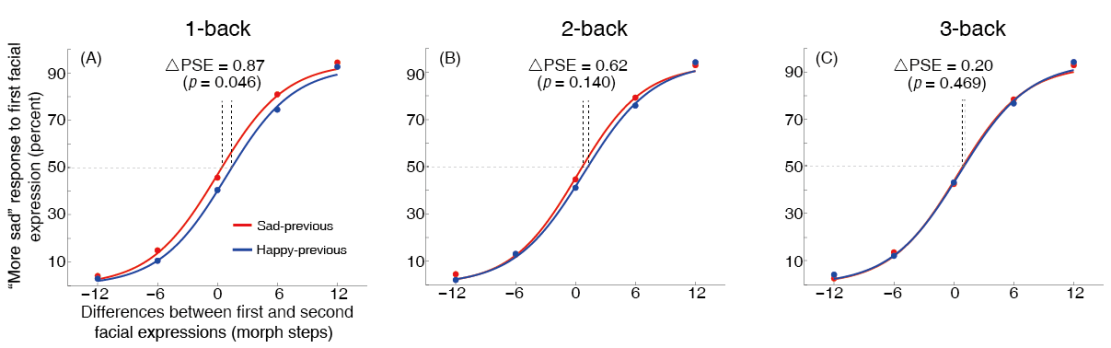


**Figure S1.** Grand average psychometric functions for Experiment 1a.

**Experiment 2**

A significant leftward shift of the happy-previous curve relative to the sad-previous was found for the 2500-ms delay duration condition (*t*(12) = 4.80, *p* < 0.001, *d* = 1.33) (Figure 2(B)), suggesting that one-back serial dependence effect of perceived emotion expression occurred. For the 50-ms delay duration condition, no serial dependence effect was observed (*t*(12) = 1.98, *p* = 0.071, *d* = 0.55)(Figure 2(A)).


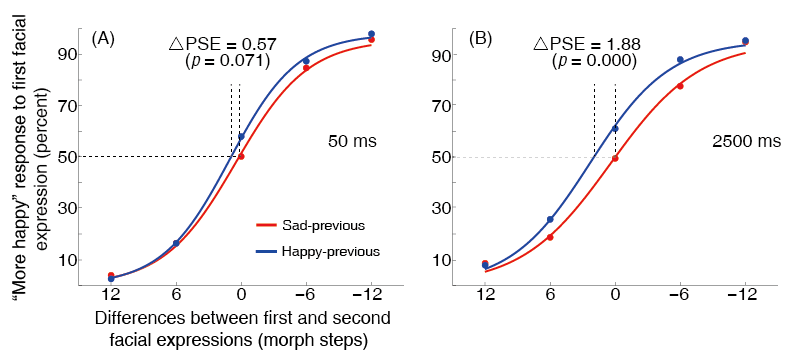


**Figure S2.** Grand average psychometric functions for the 50-ms (A) and 2500-ms (B) delay duration conditions in Experiment 2.

**Experiment 3**

For all three delay duration conditions, significant leftward shifts of happy-previous curve relative to the sad-previous were observed (50-ms: *t*(15) = 5.21, *p* < 0.001, *d* = 1.30; 1000-ms: *t*(15) = 3.81, *p* = 0.002, *d* = 0.95; 2500-ms: *t*(15) = 4.18, *p* = 0.001, *d* = 1.04), demonstrating one-back serial dependence effects (Figure 3). Although a One-Way Repeated Measures ANOVA for the three delay condition did not reach a statistical significance (*F*(_2, 30_) =2.46, *p* = 0.10, *η*2 p = 0.14), the *post-hoc* paired comparisons demonstrated that the magnitude of the serial dependence effect of the 50-ms delay duration condition was significantly greater than that of the 2500-ms delay duration condition (*p* = 0.014). An inspection of Figure S3 found that the magnitude of the serial dependence effects decreased as the delay duration of noise mask 2 increased. However, the differences between the 50-ms and 1000-ms delay duration conditions and between the 1000-ms and 2500-ms delay duration conditions did not reach a statistical significance (p > 0.10 for all two comparisons).


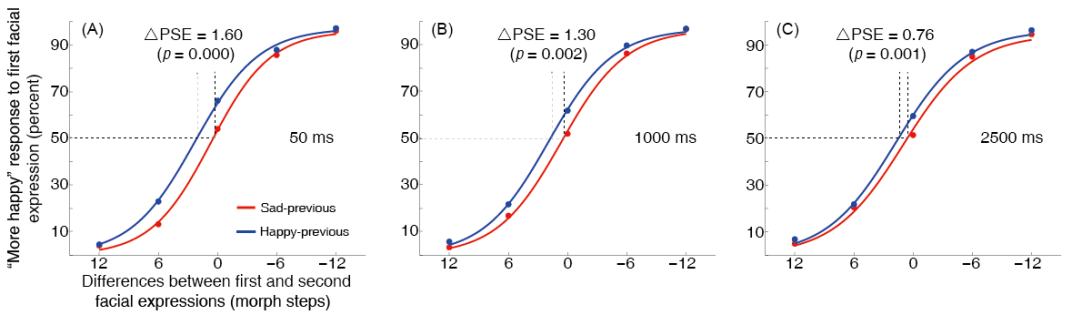


**Figure S3.** Grand average psychometric functions for the 50-ms (A), 1000-ms (B) and 2500-ms (C) delay duration conditions in Experiment 3.
